# Application of Chitosan, Chitooligosaccharide, and Their Derivatives in the Treatment of Alzheimer’s Disease

**DOI:** 10.3390/md15110322

**Published:** 2017-11-07

**Authors:** Qian-Qian Ouyang, Shannon Zhao, Si-Dong Li, Cai Song

**Affiliations:** 1 College of Ocean and Meteorology, Guangdong Ocean University, Zhanjiang 524088, China; ouyangqianqian0426@163.com; 2 College of Chemistry and Environment, Guangdong Ocean University, Zhanjiang 524088, China; lisidong2210491@163.com; 3 American Studies and Ethnicity, University of Southern California, Los Angeles, CA 90089, USA; shanmuzh@usc.edu; 4 Research Institute for Marine Drugs and Nutrition, College of Food Science and Technology, Guangdong Ocean University, Zhanjiang 524088, China; 5 Department of Psychology and Neuroscience, Dalhousie University, Halifax, NS B3H 4R2, Canada

**Keywords:** chitosan, chitooligosaccharide, neuroinflammation, oxidative stress, neuroprotective, Alzheimer’s disease

## Abstract

Classic hypotheses of Alzheimer’s disease (AD) include cholinergic neuron death, acetylcholine (ACh) deficiency, metal ion dynamic equilibrium disorder, and deposition of amyloid and tau. Increased evidence suggests neuroinflammation and oxidative stress may cause AD. However, none of these factors induces AD independently, but they are all associated with the formation of Aβ and tau proteins. Current clinical treatments based on ACh deficiency can only temporarily relieve symptoms, accompanied with many side-effects. Hence, searching for natural neuroprotective agents, which can significantly improve the major symptoms and reverse disease progress, have received great attention. Currently, several bioactive marine products have shown neuroprotective activities, immunomodulatory and anti-inflammatory effects with low toxicity and mild side effects in laboratory studies. Recently, chitosan (CTS), chitooligosaccharide (COS) and their derivatives from exoskeletons of crustaceans and cell walls of fungi have shown neuroprotective and antioxidative effects, matrix metalloproteinase inhibition, anti-HIV and anti-inflammatory properties. With regards to the hypotheses of AD, the neuroprotective effect of CTS, COS, and their derivatives on AD-like changes in several models have been reported. CTS and COS exert beneficial effects on cognitive impairments via inhibiting oxidative stress and neuroinflammation. They are also a new type of non-toxic β-secretase and AChE inhibitor. As neuroprotective agents, they could reduce the cell membrane damage caused by copper ions and decrease the content of reactive oxygen species. This review will focus on their anti-neuroinflammation, antioxidants and their inhibition of β-amyloid, acetylcholinesterase and copper ions adsorption. Finally, the limitations and future work will be discussed.

## 1. Introduction

### 1.1. Alzheimer’s Disease and Its Pathogenesis

Alzheimer’s disease (AD) is a progressive neurodegenerative disorder with memory loss, spatial disorientation, and a marked decline in intellectual capacity. The major clinical characteristics consist of extracellular senile plaques formed by the deposition of amyloid-beta (Aβ) protein, intracellular neurofibrillary tangles composed of hyper-phosphorylated tau protein, increased inflammatory response, and neuron apoptosis and death caused by oxidative stress [1,2,3]. Even though the etiology and pathogenesis of AD has not been fully elucidated [4,5,6], the hypotheses of AD pathogeneses include inflammation, oxidative stress, the accumulation of Aβ protein, lack of cholinergic neurotransmitter, the deposition of tau proteins and heavy metals, as well as the lack of neurotrophic factors, etc.

Increasing evidence suggests that chronic inflammation and oxidative stress play an important role in neurodegenerative diseases. Inflammation is the first response of the immune system to pathogens or irritation, which can produce pro-inflammatory mediators. Both peripheral and central inflammation can activate microglia, microphages in the brain, which can induce neuroinflammation. Many studies have demonstrated that chronic neuroinflammation may promote synaptic loss, cognitive dysfunction and, eventually, neuronal death [7,8,9,10,11]. As a consequence of neuroinflammation, oxidants are released and oxidative stress occurs in the brain, which are observed in the development of AD [12,13]. Reactive oxygen species [3] are the product of aerobic metabolism, and are controlled by antioxidant and intracellular enzymes such as superoxide dismutase [2], catalase, and peroxidase. In physiological conditions, ROS is considered as a signaling molecule produced at low level and in a transient manner. While excessive ROS leads to peroxidation, which can damage DNA, phospholipids, and proteins. Furthermore, these damages can reduce the oxidase activity of cytochrome C in the mitochondria and cause the metabolism disorders, eventually cell apoptosis [14]. Therefore, it becomes a new research direction to prevent and treat AD through suppressing inflammation and oxidation. 

To understand the etiology of AD and find effective treatments, several classic and new hypotheses of AD have been raised as follows: 

(1) Aβ protein accumulation: Aβ is an important part of amyloid plaques in AD brains. Mechanistically, aggregated Aβ may induce oxidative stress by causing both mitochondrial dysfunction and lipid peroxidation [14]. It has been confirmed that alpha7 nicotinic receptor (α7nAChR) on the surface of neuron cells can mediate Aβ from extracellular into intracellular neurons due to their high affinity [15]. The binding between α7nAChR and Aβ can result in aggregation of Aβ_1-42_, forming senile plaques with Aβ_1-42_ as the main component. Short-time and low-concentration of Aβ exposure do not cause the change of the signal pathways of α7nAChR, but long-time and high concentration can lead to signal pathway disorder, accompanied by learning and memory impairment [16]. The other important molecular marker of AD is miR-29, which is potentially involved in the regulation of Aβ precursor proteins (APP) and Aβ cleavage enzyme (BACE) 1 expression. In sporadic AD patients, higher BACE1 protein and lower miR-29 levels were found [17,18].

(2) Acetylcholine (ACh) deficiency: ACh is the main neurotransmitter to control learning and memory in the hippocampus. ACh can enhance memory, promote nerve conduction and facilitate long-term potentiation (LTP). Metabolic disorders of ACh can directly cause cognitive decline, such as decreased ability to study and memory. Thus, the loss of cholinergic neurons and decreased ACh levels in the brain were hypothesized to be responsible for the cognitive decline observed in AD [19]. Since ACh can be hydrolyzed by acetylcholinesterase in the brain, the symptoms of AD can be alleviated through inhibiting the activity of acetylcholinesterase [20,21].

(3) Neurofibrillary tangles (NFTs) of tau proteins: tau proteins are microtubule-associated proteins that can maintain the normal axonal transport, microtubule structure stability, phosphorylation, and dephosphorylation in a dynamic equilibrium [22,23]. With the body under pathological conditions, a variety of pathogenic factors in different pathways caused by tau protein and microtubule binding dynamic balance was disturbed. The phosphorylation rate of tau proteins is higher than the rate of dephosphorylation, which results in abnormal high levels of tau protein phosphorylation. It would cause extensive cross-linking of tubulin molecules and affect cell signal transduction, as well as ultimately cause the abnormal aggregation of tau protein and fibrosis and NFTs formation. NFTs accumulation in the degenerative neuronal somas is positively correlated with the decline of cognition and memory in AD patients in a clinical investigation [24].

(4) Increased inflammatory response: Aβ overexpression can activate glial cells such as microglia and astrocytes, which produce pro-inflammatory factors and induce neuroinflammatory cascade, leading to specific neuronal degeneration in the brain [25]. These neuroinflammatory factors, such as interleukin (IL)-1, IL-6, tumor necrosis factor (TNF)-α, and endotoxin, can reduce the early phagocytic clearance and immune surveillance of microglia cells, leading to neuroinflammatory response, nerve cell degeneration, injury and death [26]. Aβ also interacts with tau proteins to mediate the neuroinflammation to accelerate AD progression. Aβ activated glial cells and inflammatory factors have been targeted as new treatments and prevention for AD [26].

(5) Accumulation of reactive oxygen free radicals: Aβ aggregation can reduce the mitochondrial redox activity, which results in the accumulation of reactive oxygen species. The increase in free radicals in turn promotes the cleavage of β-amploid precursor protein (APP) into Aβ, thereby increasing the deposition of Aβ. Between the two there is a mutually beneficial effect, leading to nerve cells damaged in a dysfunctional vicious circle. Inhibition of oxidative stress and apoptosis of the mitochondrial signal pathway, regulation of protein apoptotic enzyme-3 activity and DNA fragment formation, a reduced Bcl-2/Bax ratio, may significantly slow down Aβ-induced neurotoxicity and neuroinflammation [27].

(6) Metal ion dynamic equilibrium disorder: Metal elements, such as iron, zinc, copper, magnesium, and manganese, exist in protein structures and modulate macromolecule syntheses of enzymes, hormones, and vitamins with other organic groups. However, if the metal elements cannot be combined with their target proteins or other ligands in an appropriate manner, it will catalyze the formation of ROS with metabolic toxicity through the Fenton reaction, attacking biomolecules and inducing cell damage. Divalent copper ions can prevent the deposition of the Aβ_42_ protein in the β-fold and clear the Aβ_42_ amyloid fibers that have been formed in the β-sheet structure, eventually preventing the formation and accumulation of brain starch plaques [28].

In addition to these, mitochondrial dysfunction, calcium toxicity, hormonal disorders, and genetic factors have also been suggested to participate in the etiology or progress of AD [29]. 

### 1.2. The Situation of Current Treatments

According to the pathogenic changes mentioned above, drugs against AD have been developed toward anti-inflammation, anti-oxidative stress, inhibiting the production of the Aβ protein, increasing ACh synthesis or protection of cholinergic neurons, and reducing tau protein abnormality. Anti-inflammatory drugs primarily are non-steroidal anti-inflammatory drugs (NSAIDs), which have been shown to delay the AD process. However, its application has been restricted in clinical treatment due to resistance and adverse reactions [30]. As mentioned above, neuronal cells are particularly sensitive to oxidative stress, but the current treatment of AD in patients with anti-oxidative drugs did not achieve satisfactory results in clinical trials.

Some BACE1 inhibitors like hydroxyethylamine, amine, arylamine, etc., are macromolecular drugs, which pass the blood brain barrier with difficulty in the treatment of AD, thereby limiting the effectiveness of the drugs [31]. Therefore, it is necessary to search for drugs with smaller molecules and higher efficacy to suppress β-amyloid. Meanwhile, some drugs suppressing the activity of acetylcholinesterase can decrease the symptoms of AD as well. *N*-methyl aspartate receptor antagonist and cholinesterase inhibitors are the uniquely FDA-approved drugs for the treatment of AD [32,33]. However, acetylcholinesterase inhibitors include tacrine, donepezil, galanthamine, rivastigmine, and all showed some limitations, such as tacrine showing some liver toxicity [34]. The pathogenesis of AD seems a synergistic action from a variety of factors and the single-target drug treatment is often not significant, while still having some side effects. Therefore, treatments with multi-target effects or combined from several medications may significantly increase clinical efficacy [35]. León et al. [35] have summarized the current therapeutic strategies based on the paradigm one-compound-various targets to treat AD.

Current therapeutic approaches for AD include antioxidants [36], vitamins [37], stem cells [38], estrogenic hormones [5], anti-hypertensive or lipid-lowering medications [39], and selective phosphodiesterase inhibitors [40], β-secretase and γ-asecretase inhibitors, tau hyperphosphorylation and intracellular NFTs inhibitors, non-steroidal anti-inflammatory drugs (NSAIDs), transition metal chelators [41], insulin resistance drugs, etanercept, brain-derived neurotrophic factors [42], etc. Many categories of synthetic compounds used to treat AD are ineffective enough but with many side effects, such as tiredness, drowsiness, sleepless, anxiety or nervousness, imbalance, etc. Thus, new drugs to treat this disease are urgent. Nowadays researchers have a great interest to study natural bioactive compounds that can treat or improve AD. Enriched marine bio-products are an important source of bioactive compounds and natural medicine. For example, an increasing number of studies have reported that chitosan (CTS) and chitooligosaccharide (COS) derived from exoskeletons of crustaceans from the ocean have potent neuroprotective properties in the treatment of AD.

### 1.3. Chitosan, Chitooligosaccharides, and Their Derivatives

Chitin, the second most abundant natural polysaccharide, is mainly found in exoskeletons of crustaceans and cell walls of fungi. There are about 10 billion tons of natural and synthetic chitin in the world [43]. CTS is a linear amino polysaccharide composed of glucosamine and n-acetyl glucosamine units and linked with β (1-4) glycosidic bonds formed by n-deacetylation of chitin, which is the only alkali polysaccharide to exist in nature. The COS, low in molecular weight and high deacetylating biodegradation products of chitosan or chitin, are extremely soluble in water due to their shorter chain lengths and free amino groups in d-glucosamine units, and easily absorbed by the human intestine [44]. Chemical modification will improve its solubility and impart new properties of the group introduced to them, which may enhance and broaden the ways to utilize CTS and COS [45]. 

In the last decade, many studies have demonstrated effects of CTS and COS, including immune regulation, antioxidant, and anti-inflammation, etc. [46,47,48,49,50,51,52,53].

## 2. Biological Activity of CTS and COS and Its Derivatives in the Treatment of AD

The neuroprotective effects of CTS and COS and the use of nanoparticles as a drug carrier through the blood-brain barrier (BBB) suggest that they are effective in the treatment of neurodegenerative diseases [54,55]. Recently, CTS, COS, and their derivatives can improve some symptoms in the development and progression of AD and be potential drugs for the prevention and treatment of AD [56]. The review below summarizes the progress of the application of CTS, COS, and their derivatives in the treatment of AD and their mechanisms. The review also aims to seek the possibility of developing CTS, COS, and their derivatives as neurotrophic drugs.

### 2.1. Anti-Neuroinflammatory

A number of studies have found the anti-neuroinflammatory activities of CTS and COS. Neuroinflammation results in oxidative stress in the synapses and mitochondria, which contributes to the neuronal and vascular degeneration in the AD brain [57,58]. Kim et al. [59] reported that the mechanism by which COS suppresses LPS-induced macrophage responses through the MAPKs signaling pathway, such as the suppression of JNK 1/2 and IκB degradation, as well as preventing NF-κB translocation into the nucleus at the cellular level. The anti-inflammatory effects of COS was also via inhibiting the activation of basophils, neutrophils, and lymphocytes [60]. Then, Kim et al. [61] used the human astrocytoma cell line as an in vitro model to explore Aβ- and IL-1β-driven inflammatory processes in AD. The results showed that high molecular weight soluble CTS could inhibit the production of pro-inflammatory cytokines such as TNF-α and IL-6 in human astrocytoma cells activated by Aβ peptide 25-35 or by IL-1β. The effects of high molecular weight soluble CTS [62] on pro-inflammatory cytokines such as TNF-α and IL-6 were evaluated by enzyme-linked immunosorbent assay and Western blotting. The secretion and expression of TNF-α and IL-6 were significantly inhibited by pretreatment with 1 and 10 μg/mL of high molecular weight soluble CTS. Moreover, the expression of inducible nitric-oxide synthase induced by Aβ_25-35_ and IL-1β was partially inhibited as well. However, Khodagholi et al. [63] focused on the anti-neuroinflammatory effect of CTS and its derivatives in NT2 neuronal cells by inducing Aβ formation through oxidative stress. The same experiment also showed that CTS exerted anti-neuroinflammatory action by up-regulation of heat shock protein 70, and inhibition of the activation of NF-κB. These findings highlight the potential role of COS as novel therapeutic agents for the prevention and treatment of AD.

### 2.2. Antioxidant Activity

As a consequence of inflammation, oxidative stress further causes neuronal apotosis and death [64,65]. CTS and COS, easily absorbed by organisms, with free hydroxyl and amino groups, can combine with superoxide free radicals as natural antioxidant, which can reduce cell oxidative damage [66,67]. Li et al. [68] have studied COS with the degree of polymerization ranging from two to 12, from which five fractions were separated by CM Sephadex C-25 column. Three antioxidant targets including hydroxyl, superoxide radical scavenging activity and reducing power were investigated. The results showed that the antioxidant activities of COS occurred in a dose-dependence manner and related to their degree of polymerization. The COS with low DP showed a better effect of scavenging hydroxyl radical and reducing power than that with the high one. By contrast, the superoxide radical scavenging activity of all tested COS was increased with DP increasing. COS can also inhibit the response of oxidative stress and neuroinflammation in the Aβ_1-42_ protein-induced model of rats, thereby significantly benefitting cognitive function [69]. In the study of COS toxic effects in Aβ_1-42_ protein-induced rat hippocampal neurons, it was found that pretreatment with COS could significantly inhibit Aβ protein-induced apoptosis, reduce the production of reactive oxygen species and lipid oxidation, and significantly block the phosphorylation of amino-terminal kinase [70].

Based on the hypothesis of increased oxidative stress induced by Aβ protein formation, Khodagholi et al. [63] had studied CTS as a protective agent against H_2_O_2_/FeSO_4_-induced cell death in the NT_2_ neural cell line. It was found that the formation of Aβ protein in NT_2_ neurons pre-treated with CTS was significantly lower than that in the control group. In the H_2_O_2_ experimental model, the Aβ protein content was decreased from 30.96 pg/mL to 22.2 pg/mL, after treatment with 0.1 and 0.5 *w*/*v* CTS. The results showed that CTS not only protected the neurons against cell death but also decreased Aβ formation. 

To improve the anti-oxidative properties of COS, COS derivatives, including gallic acid-conjugated COS [71] and sulfated COS [72], have been synthesized. Of particular interests, these COS derivatives exhibited higher anti-oxidative activity than unmodified COS in macrophages and pancreatic β cells, respectively.

### 2.3. Suppressing Aβ Cleavage Enzyme

Based on the theory of brain amyloid cascade, BACE1 is the first step to start Aβ protein production. At present, the most effective way to reduce Aβ protein is to inhibit β-secretase production, which may slow the further progression of AD [52,73,74,75]. It has been reported that injecting soluble Aβ protein into the animal brain can lead to behavior disorder and memory impairment [76,77,78,79]. Orally-administered COS at 200, 400, or 800 mg/kg doses effectively improve the learning and memory deficits in the Aβ_1-42_-induced AD model of rats. The results also showed increased activities of glutathione peroxidase and super oxide dismutase and decreased release levels of proinflammatory cytokines, such as TNF-α and IL-1. This suggested that COS attenuated the cognitive impairments via inhibiting oxidative stress and neuroinflammatory responses [69]. Another investigation of the effect of COS on oligomeric Aβ-mediated toxicity in rat primary hippocampal neurons indicated that COS remarkably prevented Aβ-induced cell apoptosis, decreased the generation of reactive oxygen species and lipid peroxidation, and blocked phosphorylation of c-Jun N-terminal kinase [70]. Recently, Dai et al. [80] also reported that COS markedly inhibited Aβ aggregation, attenuated Aβ1-42-induced neurotoxicity and reduced fibril formation in a dose-dependent manner in rat cortical neurons. 

However, different degrees of deacetylation and different molecular weight of COS may differently inhibit β-secretase. Byun et al. [81] studied the functional group of COS with different degrees of deacetylation (90%, 75%, 50%) and molecular weights (below 1 kDa, 1–3 kDa, 5–10 kDa) on β-secretase inhibitory activity. The results showed that 90% deacetylated COS passed through the 5 kDa membrane, but not the COS with 3 kDa exhibited the highest β-secretase inhibitory activity based on molecular weight of 3 and 5 kDa. 

Additionally, Eom et al. [82] synthesized eight phenolic acid conjugated CTS with hydroxybenzoic acid and hydroxyl phenyl acrylic acid to evaluate their inhibitory activity against BACE, and found that caffeic acid conjugated CTS is a novel potential BACE inhibitor that can be used as a potential drug to improve AD. The IC 50 values of these COS derivatives are comparable to a positive control drug galatamine [83], suggesting that these COS derivatives may be as efficacious as the positive control drug in the treatment of AD. By another way, the delivery system of CTS-coated and uncoated solid lipid nanoparticles to obtain an efficient and optimal nose-to-brain transport of BACE1 sinRNA was designed, potentially useful in the treatment of AD [84]. Taken together, these results confirm that CTS plays a role in inhibiting Aβ protein levels, thereby relieving the process of AD.

### 2.4. Acetylcholinesterase Inhibitory Activity

COS are a type of natural carbohydrates with non-toxicity and high biological activity, which are also easily absorbed by the body due to their soluble properties. Several studies showed that COS and its derivatives, including dimethyl-COS and diethyl-COS, exhibited inhibitory activity on acetylcholinestsrase [85], while diethyl-COS showed the highest inhibitory activity because of its hydrophobicity. Meanwhile, the inhibitory activity of acetylcholinesterase could be enhanced with an increase to the degree of deacetylation of COS. These results suggest that COS and its derivatives have the potential to be applied as new and non-toxic AChE inhibitors for preventing AD [85]. Furthermore, nasal galantamine hydrobromide/CTS complex nanoparticles could provide therapeutic potential for managing AD through assessing brain AChE protein levels and activity in rat brains [86].

### 2.5. Adsorption of Copper Ions

Copper ions play a very important role in cell physiology and serve as a cofactor for many enzymes, such as cytochrome oxidase, dopamine hydroxylase, and amidase. Copper accumulation also plays an important role in the pathogenesis of AD by inducing oxidative stress and associated neuronal damage [87]. Copper ions are reducible, which can catalyze the production of reactive oxygen species. Thus, its accumulation will cause neuronal oxidative stress and neuronal toxicity [68]. Therefore, dynamic equilibrium disorder in copper ions is another key factor leading to neurodegenerative diseases, such as AD. Since COS contain hydroxyl, amino, and acetamido groups, they can chelate with metal ions, including copper ions to form complexes [88], thereby reducing their toxic effects [88]. Xu et al. [68,89,90] studied the protective effect of COS on copper ion-induced neurotoxicity. The administration of 50 µM of copper chloride can cause cell damage, but after treating with COS (molecular weight of 1500, degree of deacetylation of 90%, and below 1 mg/mL), cell viability was increased in a dose-dependent manner. Especially when the concentration of COS was at 0.4 mg/mL, the increase of cell viability is nearly 40%, which is much higher than the changes after other COS concentration administrations. There was no toxic effect on cell viability after COS treatment [90]. According to these findings, the release of lactic dehydrogenase and the content of reactive oxygen species in the cells were further determined. The results showed that COS as a neuroprotective agent could reduce cell membrane damage and decrease the content of ROS caused by copper ions. Taken together, COS, on the one hand shows antioxidant properties, and on the other hand, can form a complex with copper ions. These findings suggest that COS can be used as a neuroprotective agent for the treatment of AD and other neurodegenerative diseases.

### 2.6. Neuroprotective Functions

One of the pathological changes of AD is neuronal apoptosis. Thus, neuronal protection is another key point to prevent and treat AD [91]. Animal experiments showed that COS can protect and repair peripheral nerve injury in rabbits [92]. The studies have investigated the possible benefits of treatments with COS on nerve regeneration after crush injuries to peripheral nerves. The rabbits with a crushed common peroneal nerve were treated by a daily intravenous injection of 1.5 or 3 mg/kg body weight of COS or an identical volume of saline (as the control) for a six-week period. At the end of COS treatment, electrophysiological assessments, Meyer’s trichrome and Masson trichrome staining, and transmission electron microscopy were used to evaluate the regeneration of injured common peroneal nerve and the atrophy of the tibialis posterior muscle. The results showed that the action potentials of compound muscles, the number of regenerated myelinated nerve fibers, the thickness of regenerated myelin sheaths, and the cross-sectional area of tibialis posterior muscle fibers were all significantly improved in the group with COS treatment in a dose-dependent pattern. This study demonstrated that COS accelerated peripheral nerve regeneration after crush injury to rabbit common peroneal nerves. Another similar experiment by Jiang et al. [93] reported that COS promoted nerve cell differentiation like a nerve growth factor in the rat model of sciatic nerve crush injury. The reason is that COS can enhance the expression of neurofilament proteins and cadherin in cells and promote neuronal differentiation, synaptic growth, and nerve cell adhesion. 

**Figure 1:**
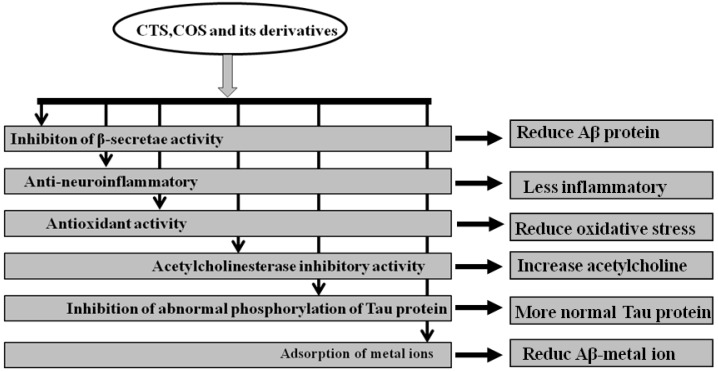
A summary of the mechanisms by which chitosan (CTS), chitooligosaccharide (COS), and their derivatives treated Alzheimer’s disease (AD).

Furthermore, COS and their derivatives were found to inhibit apoptosis related molecular changes. Koo et al. [94] have found that high molecular weight water-soluble (WSC) CTS was able to prevent serum-starved human astrocytoma cells from undergoing apoptosis. It is well known that glutamate accumulation in the central nervous system (CNS) and the overexpression of glutamate receptors-induced excitotoxicity are other pathogenic triggers of AD and CNS injuries. However, COS can reduce hippocampal neuronal apoptosis induced by glutamate [95]. In the vitro drug release profile and in vivo for sampling of blood from donepezil-administered rats, Keshireddy et al. [96] have further reported that rapidly disintegrated donepezil hydrochloride oral thin films were successfully formulated by incorporating microsized CTS thin film, nanosized CTS thin film, and CTS nanofiber in the treatment of AD. In summary, the mechanism by which CTS, COS, and their derivatives treated AD may be through the inhibition of β-secretase activity, anti-neuroinflammation, antioxidant activity, inhibition of acetylcholinesterase activity, and abnormal phosphorylation of tau protein, and adsorption of metal ions is shown by Figure 1. 

### 2.7. Other Activities

Nanoparticles are a drug delivery system that can change the pharmacokinetic properties of drugs, especially the surface-modified polymer nanoparticles, which can act on the central nervous system through BBB [18]. As natural mucoadhesives with good biocompatibility, CTS and COS are commonly used in mucosal administration of drug [97]. As a carrier of drugs, CTS and COS can also transport bioactive substances through the BBB to nerve cells at the molecular level. Pereira et al. [98] have verified that CTS/pre-miR-29b and polyethylenimine/pre-miR-29b systems efficiently delivered pre-miR-29b to N2a695 cells, thus reducing the human BACE1 protein expression and Aβ42 levels. A study has demonstrated that CTS can be used as a carrier material and can significantly enhance the delivery efficiency of anti-AD drugs from the nose to the brain. For example, piperine is a plant neuroprotective drug used in the treatment of AD due to its acetylcholinesterase inhibition and antioxidant effects. However, oral administration of piperine is not feasible because of its hydrophobicity and pre-metabolic property. With CTS nanoparticles loaded with piperine in the nasal cavity, it can be effectively applied to the brain as a kind of targeted therapy. Meanwhile, monodisperse CTS nanoparticles can significantly alleviate the stimulation of piperine on the nose without toxic effects on the brain. In the treatment of AD, soluble CTS nanoparticles were also found to safely and efficiently deliver piperine to targeted sites and multiply, which can reduce the oral dose by 20 times [99]. In this paper, Kaur et al. [100] reported that the synthesis and stability studies of novel CTS engineered thyrotropin-releasing hormone encapsulation poly (lactide-*co*-glycolide) showed the potential membrane penetrating and effective delivery of neuropeptides, which were used in the treatment of brain/spinal injury and certain CNS disorders, including schizophrenia, AD, depression, etc. Rosmarinic acid-loaded polyacrylamide-chitosan-poly (lactide-*co*-glycolide) nanoparticles were grafted with cross-reacting material 197 and apolipoprotein E to pass across the BBB and rescuing neurodegeneration. The change can be a promising formulation to deliver rosmarinic acid to insulted neurons in the pharmacotherapy of AD at the cellular level [101]. In in vitro permeation studies, a novel approach for preparation of a six-day transdermal drug delivery system as treatment for mild to moderate AD was considered as an alternative for one-week application of six Exelon patches [102]. Based on CTS of sodium alaginate, Yalcin et al. [103] found that the treatment with HP-CD microspheres against Aβ_1-42_ decreased the levels of lipid peroxidation and reactive oxygen species production. The results indicated that nasally-administered spray-dried HP-CD microspheres were able to provide protection against Aβ_1-42_-induced neurotoxicity in AD model rats. In another experiment, ceria-containing carboxymethyl CTS-coated hydroxyapatite-based galantamine nanocomposites effectively treated AD symptoms in an ovariectomized albino-rat model [104]. *Nigella sativa* with CTS-modified particles was also found to enhance treatment benefits for AD at the cellular level [105].

## 3. Limitations and Future Studies

In this paper, we have introduced the neuroprotective effect of CTS, COS, and their derivatives for the treatment of AD, including their functional characteristics and the improvement on the pathologic factors of AD. These results suggest that CTS, COS, and their derivatives have great potential as promising drugs to treat neurodegenerative diseases like AD. However, the effects of CTS, COS, and their derivatives on a chronic inflammation-induced AD model, AD transgenic models, and their effect on neurotrophic factors have not been properly evaluated. Furthermore, in vitro research about the anti-neuroinflammatory effects of CTS and its derivatives on microglial activation-induced neuronal apoptosis and the interaction among microglia, astrocytes, and neuronal cells are needed. In an in vivo study, the improvement of CTS, COS, and their derivatives on awareness, learning, and memory, as well as emotion in AD animal models, should be studied. To date, research on CTS and COS has also lacked investigation into memory-related electrophysiological activities and learning and memory related neurotransmitter functions. Therefore, future studies should focus on the treatment mechanism of CTS, COS, and their derivatives in the models and aspects mentioned. Whether CTS, COS, and their derivatives in regulating transcription factors and signal molecules have some relationship with the composition of neurovirulence or not also needs to be determined. Hadwiger et al. [106,107,108] reported that CTS could enter the plant nucleus, activate plant defense genes to resistant infection, reduce DNA damage, and inhibit germination. Since immune dysfuntion and inflammation are the contributors to AD, the above findings may suggest a new research direction at immuno-molecular level. 

In addition, since acetylcholinesterase inhibitors can moderately improve the symptoms of AD and delay memory decline, it is necessary to study how these compounds inhibit acetylcholinesterase activity in cellular and in animal models of AD to further elucidate the neuroprotective effects of CTS and COS. The molecular mechanism of CTS- and COS-treated Alzheimer’s disease is also important. There is not enough data to reveal the molecular mechanisms by which CTS and COS improve the pathological changes of AD, such as anti-inflammatory, anti-apoptosis, and anti-oxidative pathways. Additionally, those receptors and molecular markers related to the etiology of AD, such as the expressions and functions of the alpha7 nicotinic receptor and mirRNA, should be evaluated in the future.

## Abbreviations

α7nAChR	Αlpha7 nicotinic receptor
Aβ	Amyloid-beta
ACh	Acetylecholine
AChE	Acetylcholinesterase
AD	Alzheimer’s Disease
APP	β-amyloid precursor protein
BACE	Amyloid-beta Cleavage Enzyme
BBB	Blood-Brain Barrier
CNS	Central Nervous System
COS	Chitooligosaccharide
CTS	Chitosan
IL	Interleukin
LTP	Long Time Potentiation
NFTs	Neurofibrillary Tangles
NSAIDs	Non-Steroidal Anti-inflammatory Drugs
ROS	Reactive Oxygen Species
SOD	Superoxide Dismutase
TNF	Tumor Necrosis factor
WSC	Water-Soluble Chitosan
